# Intelligence

**Published:** 1828-05

**Authors:** 


					INTELLIGENCE.
MONTHLY REPORT OF PREVALENT DISEASES.
The cold weather of the last month has brought with it a plentiful supply of
rheumatism and catarrhs, and some more acute inflammations. The first of
these has been, in some cases, extremely obstinate, running into the chronic
form, and requiring lengthened treatment. The inflammations have had
their seat chiefly in the mucous membranes; and one or two laryngeal affec-
tions which we have witnessed have been very severe, only yielding to active
bleeding (chiefly local) and to the free use of mercury. Fever, too, has
presented itself, generally in a mild, but sometimes in a more aggravated
l'orm, with a good deal of abdominal affection.
METEOROLOGICAL JOURNAL,
From March 2Mh, to April 2Oth, 1828.
By Messrs. Harris and Co. Mathematical Instrument Makers, 50, High Holboru.
20
21
22
23
24
25
26
27
28
29
SO
31
April
?I
3
4
5
6
7
8
9
10
11
12
13
14
15
16
17
18
19
.40
o
Thermom.
Barometer.
s
29.33
29 00
29.17
29.30
29.57
29.66
29.75
29.48
29.56
29.70
29.88
30.15
30.17
29.92
29.97
29.81
29.66
29.49
29.24
29.22
29.24
29.27
29.61
29.53
29.37
29.65
29.63
29.45
29.30
29.22
29.42
s
29.07
29.00
29.28
29.43
29.63
29.75
29.63
29.33
29.63
29.76
30.04
30.22
30.00
29.94
29.86
29.84
29.56
29.47
29.31
29.10
29.34
29.41
29.62
29.47
29.50
29.71
29.51
29.24
29.24
29.34
29.63
94
Winds.
W
wsw
w
NW
N
NE
NE
KSE
NNE
NE
NE
SE
SSE
ENE
N
N
WNW
NE
SE
SE
SW
SW
WNW
W
SW
w
wsw
wsw
SW
SSW
ESE
W
w
WNW
NW
N
NE
S
E
NE
NW
SE
SSE
S
ENE
NE
N
ENE
.SE
NE
NE
SW
W
W
w
SW
wsw
wsw
SW
SW
SW
E
Atmospheric Varaitions.
9 a.m. 2p.m. 10p.m
Cloudy
Fine
Foggy
Rain
Cloudy
Fair
Foggy
Fine
Cloudy
Kaiu
Cloudy
Haiu
Fine
Cloudy
Fair
Rain
Fair
Hail
Raiu
Fair
Sbow'ry
Cloudy
Raiu
Fair
Rain
Fair
Fine
Cloudy
Fair
Rain
Fair
Rain
Cloudy
Fair
Rain
Sleet
Cloudy
Fine
Hain
Cloudy
Fine
Cloudy
Fine
Cloudy
Rain
C loudy
Fair
Clondy
Fair
Cloudy
Rain
Fine
Cloudy
Fine
Cloudy

				

